# An Application for Pruritus Vulvæ

**Published:** 1886-11

**Authors:** 


AN APPLICATION FOR PRURITUS VULVÆ.—

| | |
|---|---|
| Glycerite of Starch, - - - | 30 parts. |
| Zinc Oxide, - - - - | 6 " |
| Potassium Bromide, - - - | 10 " |
| Ext. of Indian Hemp, - - - | 2 " |

Precede the application by a hot hip-bath.—*New York Med. Jour.*

---

"A MAN who had sore eyes went to a horse doctor for relief. The doctor applied to his eyes an ointment he was accustomed to use on horses. The man became blind, and sued the doctor, but judge acquitted the horse doctor, on the ground that if the man had not been an ass he would never have applied for relief from a horse doctor."—*Ex.*

---

AN ITALIAN writer has collected from ancient and mediæval literature seventy-three cases of entire abstinence from food for periods varying from three days to as many years. There were Tanners and Succis many centuries ago.

